# Adherent-invasive *Escherichia coli* associated with granulomatous colitis and extraintestinal dissemination in a Sphynx cat

**DOI:** 10.1177/03009858231162204

**Published:** 2023-03-23

**Authors:** Laura Tuomisto, Ravi Kant, Anna-Mariam Kiviranta, Kukka-Maaria Helkiö, Tarja Sironen, Antti Sukura, Rebecca P. Wilkes, Kristel Kegler

**Affiliations:** 1 University of Helsinki, Helsinki, Finland; 2 Purdue University College of Veterinary Medicine, West Lafayette, IN

**Keywords:** AIEC, adherent-invasive *Escherichia coli*, cat, extraintestinal dissemination, granulomatous colitis

## Abstract

This case report describes a case of granulomatous colitis (GC) associated with adherent-invasive *Escherichia coli* (AIEC) with extension to cecum and ileum and dissemination to multiple lymph nodes, the spleen, and brain in a 10-year-old, male Sphynx cat. The cat had an episode of diarrhea 4 months prior to consultation due to sudden blindness. Signs rapidly progressed to ataxia, seizures, and death. Gross and histologic findings were consistent with granulomatous inflammation in all affected organs. *In situ* hybridization confirmed the presence of intracellular *E. coli* within enterocytes and infiltrating macrophages, and whole genome sequencing identified virulence traits commonly linked to AIEC strain. This is the first characterization of GC in a cat associated to AIEC resembling the metastatic form of Crohn’s disease in humans and GC of dogs. Extraintestinal involvement might provide evidence of the ability of AIEC to promote granulomatous inflammation beyond the gut.

Adherent-invasive *Escherichia coli* (AIEC) is a pathovar lacking virulence factor-encoding genes typically present in other pathogenic *E. coli* species.^1,5,16^ This strain is implicated in the etiopathogenesis of inflammatory bowel disease (IBD) of humans, particularly Crohn’s disease (CD), and in granulomatous colitis (GC) of Boxers and French Bulldogs.^6,10,19^ To date, only 3 cases of GC in cats presumed to be caused by *E. coli* based on biopsy findings and response to antibiotic therapy are reported.^12,15,17^ The diagnosis of CD and GC is based on characteristic mucosal ulceration accompanied by periodic acid-Schiff (PAS)-positive macrophages/histiocytes extending into the submucosa. Inflammation is described only in the colon of cats, while in people and dogs, the ileum, cecum, and mesenteric lymph nodes are compromised in variable degrees.^12,13,15,17,19^ Extraintestinal involvement, referred to as metastatic CD, occurs in up to 36% of affected humans with cutaneous and mucocutaneous granulomas being among the most common manifestations.^9,20^ The role of AIEC in metastatic CD has not been explored and a metastatic form is not reported in dogs with GC.

A 10-year-old, neutered male Sphynx cat presented to the Veterinary Teaching Hospital, University of Helsinki, with a 1-week history of sudden blindness. The cat had mild diarrhea and weight loss 4 months prior to consultation, and an unremarkable abdominal ultrasound 2 weeks before the onset of blindness. On clinical examination, peripheral lymphadenomegaly was observed. Ophthalmologic evaluation showed an absent menace response and marked pupillary light and dazzle reflexes, suggesting an underlying neurologic deficit. Neurologic and cranial nerve examinations identified bilateral reduction of nasal mucosal response. Orientation and gait were normal. Neuroanatomical localizations of the deficits included the optic chiasm and forebrain. Serum biochemistry revealed hyperglobulinemia and hematology was unremarkable. IDEXX SNAP FIV/FeLV Combo Test for feline immunodeficiency virus and feline leukemia virus, and a serologic test for *Toxoplasma gondii* were negative. Four days later, neurologic signs progressed to ataxia, circling, and generalized epileptic seizures, and the cat died at home.

At necropsy, the wall of the colon, cecum, and distal ileum was thickened by white-tan, firm coalescing nodules (Fig. 1a). The overlying mucosa was multifocally ulcerated. The ileocecal, mediastinal, tracheobronchial, submandibular, prescapular, and popliteal lymph nodes were enlarged and multinodular. The spleen had multifocal, variably sized, white-tan nodules. The meninges covering the ventral olfactory bulb, the frontal lobe, and extending to the rostral medulla oblongata including the piriform lobes were irregularly thickened and edematous. The optic tract and optic chiasma were effaced and unapparent. The oculomotor (III), trochlear (IV), and trigeminal (V) nerves were thickened and firm. There were no other remarkable gross findings. At this point, differential diagnoses included disseminated granulomatous disease caused by mycobacterial or fungal infection, lymphoma, and feline infectious peritonitis (FIP). Representative samples were placed in 10% neutral buffered formalin, paraffin embedded, prepared as 5 µm sections, and stained with hematoxylin and eosin.

**Figure 1.**
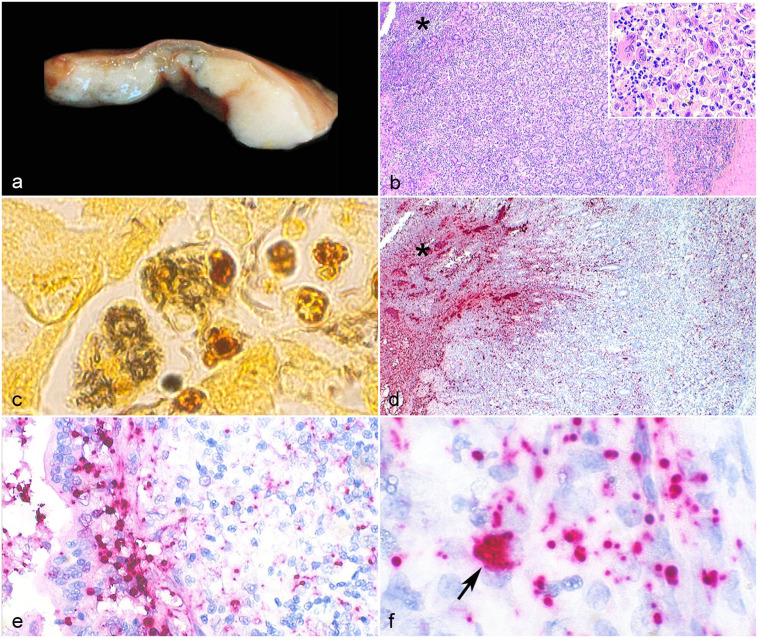
Granulomatous colitis associated with adherent-invasive *Escherichia coli*, colon, cat. (a) The mucosa and submucosa are markedly thickened by white tan and firm coalescing nodules. The overlying mucosa is ulcerated. (b) Section of the ileocolic junction showing mucosal necrosis (asterisk) and inflammation dissecting the lamina propria and extending deep into the submucosa. Inset: Inflammatory infiltrate consisting predominantly of foamy and epithelioid macrophages; small numbers of multinucleated giant cells; and few neutrophils, lymphocytes, and plasma cells. Hematoxylin and eosin (HE). (c) Numerous rod-shaped bacteria are identified within the cytoplasm of 2 macrophages with Warthin Starry stain. (d) *E. coli 16S* rRNA probe hybridization (red) is present throughout the necrotic mucosa (asterisk), crypt lumen, and affected lamina propria and submucosa. (e) Probe hybridization (red) is prominent within the cytoplasm of enterocytes, and (f) intracellularly within macrophages (arrow). *In situ* hybridization (ISH), B-*E.Coli*-*16S*rRNA probe. AIEC, adherent-invasive *Escherichia coli.*

Histologically, granulomatous inflammation consisting of macrophages with foamy cytoplasm, epithelioid macrophages, and few multinucleated giant cells dissected the lamina propria and extended into the submucosa of the colon, cecum, and ileum (Fig. 1b). Lymphocytes, plasma cells, and neutrophils were present in smaller numbers. The mucosa was multifocally necrotic and covered by bacterial biofilms. Lymph node and splenic lesions were compatible with granulomatous inflammation. Sections of the brain had diffuse and predominantly histiocytic infiltration in the ventral leptomeninges with complete obliteration of the optic chiasm, optic tract, hypophysis, trigeminal nerve, and ganglia.

Immunohistochemistry (IHC, Supplemental Materials) confirmed a predominance of Iba-1-positive macrophages in the affected intestine. B-lymphocytes (CD45r) and T-lymphocytes (CD3) were present in smaller numbers. Anti-feline coronavirus (FCoV) IHC for FIP was negative in the ileocolic junction, ileocecal lymph node, spleen, and brain. FCoV real-time polymerase chain reaction (RT-PCR) was performed on formalin-fixed, paraffin-embedded from the spleen and brain (Supplemental Materials) and was negative. Conventional and modified Ziehl-Neelsen, Grocott’s methenamine silver and PAS stains ruled out acid fast-positive bacterial and fungal infections in the ileocolic junction, ileocecal, and submandibular lymph nodes, spleen, and brain. Variable numbers of macrophages and epithelioid macrophages had PAS-positive intracytoplasmic granules (Supplemental Figure S1).

Sections of ileocolic junction, ileocecal and submandibular lymph nodes, spleen, and brain were stained with Gram and Warthin Starry (WS) stains. The intestinal biofilm consisted of Gram-negative and WS positive rod-shaped bacteria. Small numbers of Gram-positive and WS-negative rods clustered in the lumen. Extracellular and intracellular WS-positive rods were present in enterocytes and macrophages in the mucosa and submucosa (Fig. 1c), and within macrophages in the lymph nodes, spleen, and brain. There were no coccoid or rod-shaped Gram-positive bacteria in the lymph nodes, spleen, or brain. *In situ* hybridization (ISH) using an RNAscope probe (Advanced Cell Diagnostics, Newark, CA, USA) targeting *E. coli 16S* rRNA (B-*E. Coli*-*16S*rRNA, cat. 433291) and RNAscope 2.5 HD Reagent Kit-Red (cat. 322350) was performed according to the manufacture’s protocol (Supplemental Materials). *In situ* hybridization punctuate reaction was present in the biofilm, lamina propria, and submucosa of the ileocolic junction (Fig. 1d). Multifocally, superficial enterocytes and crypt epithelial cells had positive hybridization in the apical surface, and enterocytes (Fig. 1e) and macrophages (Fig. 1f) had intracellular labeling. *In situ* hybridization also demonstrated *E. coli 16S* rRNA within macrophages in the ileocecal and submandibular lymph nodes, spleen, and brain.

*E. coli* was isolated from routine bacterial culture on frozen samples of spleen and brain, since intestines were not available for culturing. *Salmonella* and *Mycobacteria* did not grow in specific cultures. DNA extraction was performed on a pure *E. coli* colony using a commercial extraction kit (Promega, Madison, Wisconsin, USA), and was sent for whole genome sequencing (Illumina platform). Determination of virulence factors, *fimH* allele and *in silico* grouping^1^ was performed as described in Supplemental Materials. The genome of this isolate was named *E. coli* 2008 and deposited in GenBank under the accession number JAMQJV000000000. The virulence genes detected are listed in Table 1. Fifty-two (52) genes were related to antibiotic resistance (Supplemental Table S1). Toxins and superantigen genes were not present.

**Table 1.** Virulence genes detected in the *E. coli* isolate from this cat.

Virulence Factor	Percent Identity	Protein Function
*Air*	100	Enteroaggregative immunoglobulin repeat protein
*chuA* ^a^	100	Outer membrane hemin receptor
*cvaC*	100	Microcin C
*eilA*	99.94	Salmonella HilA homolog
*etsC*	100	Putative type I secretion outer membrane protein
*fyuA*	100	Siderophore receptor
*Gad*	100	Glutamate decarboxylase
*hlyF*	100	Hemolysin F
*iron*	99.95	Enterobactin siderophore receptor protein
*irp2* ^a^	100	High molecular weight protein 2 non-ribosomal peptide synthetase
*Iss*	100	Increased serum survival
*iucC*	100	Aerobactin synthetase
*iutA*	100	Ferric aerobactin receptor
*kpsE*	99.56	Capsule polysaccharide export inner-membrane protein
*kpsMII_K1*	99.74	Polysialic acid transport protein; Group 2 capsule
*lpfA*	100	Long polar fimbriae
*mchF*	100	ABC transporter protein MchF
*neuC*	100	Polysialic acid capsule biosynthesis protein
*ompT*	100	Outer membrane protease (protein protease 7)
*papC*	99.92	Outer membrane usher P fimbriae
*terC*	98.75	Tellurium ion resistance protein
*traT*	100	Outer membrane protein complement resistance
*Tsh*	100	Temperature-sensitive hemagglutinin

Abbreviation: ABC: Bacterial ATP-Binding Cassette; AIEC, adherent-invasive *Escherichia coli.*

a Virulence factors significantly associated with AIEC strains.^2^

The identification of AIEC is challenged by lack of specific molecular markers linked to this pathovar. Several virulence factors have been associated with AIEC pathogenic properties; however, they are not exclusive to the strain.^2,16^ Hence, defining AIEC requires a combination of genomic profiles and phenotypical traits including (1) absence of common virulence factors found in other pathogenic *E. coli*, and (2) the ability to adhere to and invade epithelial cells and to survive within macrophages.^5,10,11,16^ A distinct feature of AIEC strains isolated from humans with CD is that they all have virulence genes related to iron uptake.^2^ These iron transporters genes are *chuA, irp2*, and *fhuD. chuA* and *irp2* were found in the isolate from this cat. The *fhuD* gene is common among pathogenic and non-pathogenic *E. coli* strains^2,16^ and is not included in VirulenceFinder; however, based on annotation of this genome, it also has *fhuD*. The *chuA* and *irp2* genes are less common among diarrheagenic and commensal *E. coli* strains.^2^*FimH* mediates adhesion of AIEC strains to apical surfaces of ileal epithelium, specifically to CEACAM6, which is overexpressed in patients with CD.^2,18^*FimH* also allows epithelial cell invasion.^18^ Based on FimTyper results, the isolate from this cat has *fimH* type *fimH27*. Additionally, the *ipfA* (long polar fimbriae) gene present in this isolate is considered a key virulence factor in AIEC for adherence to M cells lining Peyer’s patches.^2,16^ Studies have shown that *E. coli* strains associated with extraintestinal infections usually belong to phylogeny groups B2 or D. These groups tend to be overrepresented in AIEC from CD.^2,4,16^ The *E. coli* from this cat is a group D strain based on *in silico* analysis,^1^ and phylogenetically it groups with a known AIEC strain (Supplemental Figure S2). Robust biofilm formation, as observed in this case, is another pathogenic trait that distinguishes this pathovar from non-pathogenic *E. coli*.^14^ Additional tests including gentamicin sensitivity and *in vitro* characterization of adhesion and invasion were not conducted in this case. Nonetheless, whole genome sequencing results combined with *in situ* detection of *E. coli* within the cytoplasm of enterocytes and macrophages allows classifying this isolate as an AIEC strain.^1,2,10,16^

The gross and histologic findings in this cat resemble CD in humans and GC of dogs. Additionally, the grouping of this isolate with known human and canine AIEC strains supports the hypothesis that AIEC could be associated with granulomatous colitis across species.^6,10,13,19^ A potential bidirectional transmission between man and animals is speculated, but not yet proven.^13^

Loss of gut homeostasis seems to be a key event promoting AIEC invasion and disease perpetuation.^3,5^ Recent investigations show that AIEC primary targets M cells and that lymphoid follicles are the initial site of inflammation.^2,3,16^ This unique feature is reinforced in this case as intestinal lesions were restricted to the ileum, cecum, and colon, which contains prominent Peyer`s patches. Dissemination of AIEC to mesenteric lymph nodes occurs in people and dogs,^6,10,19^ but extension to distant lymphoid organs and the brain, mimicking a metastatic disease with a fatal outcome, has not been reported before. It remains to be determined whether this trait is specific for this isolate, or if it represents a more complex host–pathogen interaction due to individual susceptibility in this cat. Investigation of genetic defects in the *CD48/SLAM* gene family on chromosome 38, which is implicated in selective sensing and killing of *E. coli* in human IBD and GC of dogs^8^ was not conducted in this case. Additionally, the presence of AIEC in affected organs distant to the gut raises the question of possible involvement of this pathovar in the pathogenesis of metastatic CD.

Malakoplakia and FIP should be considered as primary differential diagnoses in this case. Malakoplakia is a granulomatous condition featuring PAS-positive macrophages, with or without von Kossa-positive inclusions, and intracellular *E. coli*.^7^ Unlike this case, malakoplakia tends to be organ/system restricted without dissemination and lacks epithelioid macrophages or multinucleated giant cells.^7^ FIP was ruled out by RT-PCR and IHC; however, detecting FCoV can be challenging if the viral load is low. Unfortunately, frozen organs were not available for RT-PCR. To strengthen the case workup, we performed *E. coli* ISH on 3 selected FIP cases including 1 central nervous system (CNS) form. *E. coli* hybridization was restricted to the lumen of intestinal sections and was not present within FIP lesions (data not shown). Together, these findings suggest no apparent link between FIP and AIEC-associated granulomatous disease.

In conclusion, AIEC should be considered a differential etiology in cats with granulomatous inflammation in the colon, cecum, and ileum with or without dissemination. Additional cases and experimental infection are needed to confirm AIEC as the cause of GC in cats and to unveil the pathogenesis of metastatic manifestation.

## Supplemental Material

sj-pdf-2-vet-10.1177_03009858231162204 – Supplemental material for Adherent-invasive Escherichia coli associated with granulomatous colitis and extraintestinal dissemination in a Sphynx cat Click here for additional data file. Supplemental material, sj-pdf-2-vet-10.1177_03009858231162204 for Adherent-invasive Escherichia coli associated with granulomatous colitis and extraintestinal dissemination in a Sphynx cat by Laura Tuomisto, Ravi Kant, Anna-Mariam Kiviranta, Kukka-Maaria Helkiö, Tarja Sironen, Antti Sukura, Rebecca P. Wilkes and Kristel Kegler in Veterinary Pathology

sj-xls-1-vet-10.1177_03009858231162204 – Supplemental material for Adherent-invasive Escherichia coli associated with granulomatous colitis and extraintestinal dissemination in a Sphynx cat Click here for additional data file. Supplemental material, sj-xls-1-vet-10.1177_03009858231162204 for Adherent-invasive Escherichia coli associated with granulomatous colitis and extraintestinal dissemination in a Sphynx cat by Laura Tuomisto, Ravi Kant, Anna-Mariam Kiviranta, Kukka-Maaria Helkiö, Tarja Sironen, Antti Sukura, Rebecca P. Wilkes and Kristel Kegler in Veterinary Pathology
