# Supplementary material for: Adherent-invasive Escherichia coli associated with granulomatous colitis and extraintestinal dissemination in a Sphynx cat
Source: Vet Pathol. 2023 Mar 23;60(3):336–40. doi: 10.1177/03009858231162204 (PMC10150253; doi:10.1177/03009858231162204)
Supplement: sj-pdf-2-vet-10.1177_03009858231162204 – Supplemental material for Adherent-invasive Escherichia coli associated with granulomatous colitis and extraintestinal dissemination in a Sphynx cat [file sj-pdf-2-vet-10.1177_03009858231162204.pdf]

Supplemental Materials

Adherent-invasive *Escherichia coli* associated with granulomatous colitis and extraintestinal dissemination in a Sphynx cat.

Tuomisto et al.

Supplemental Figure S1. Granulomatous colitis associated to AIEC, colon, cat. Section of the ileocolic junction showing numerous macrophages with intracytoplasmic PAS-positive granules. PAS stain.

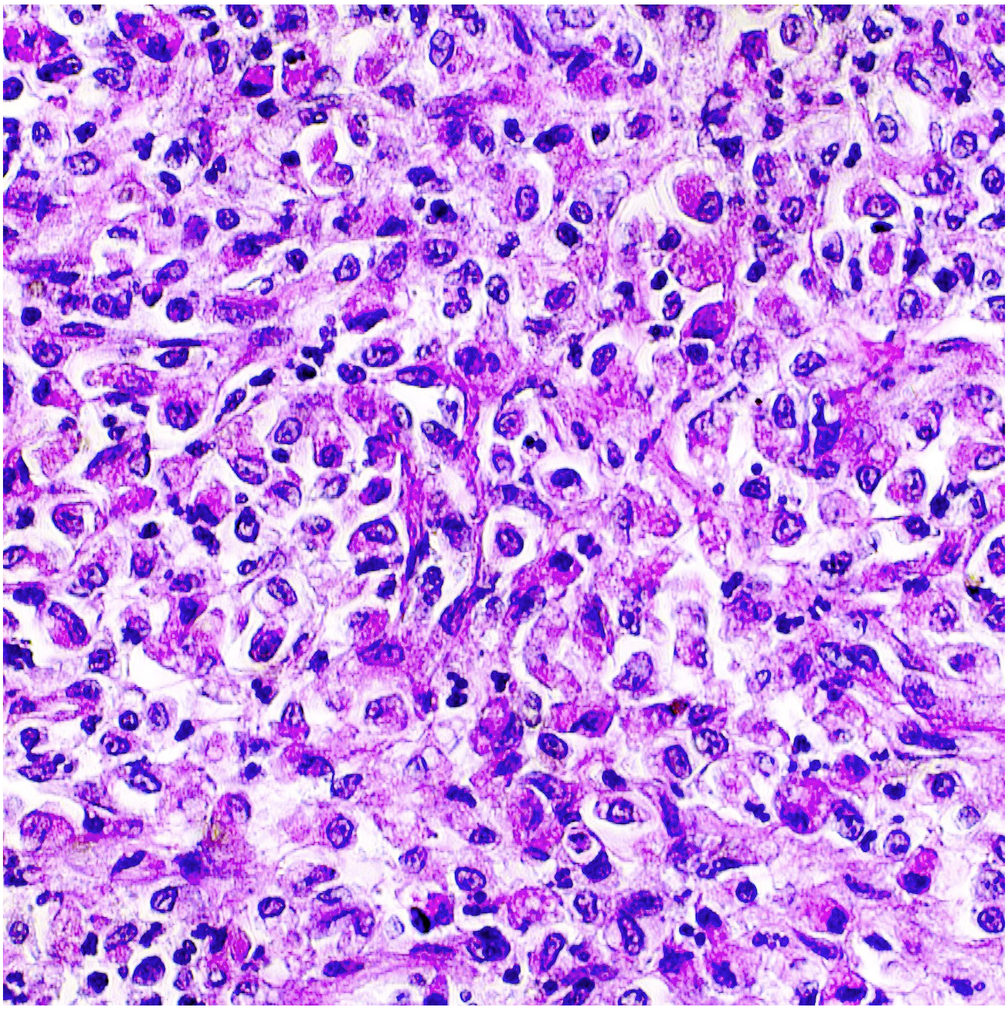

Supplemental Figure S2. Phylogenetic tree containing pathotypes of *E. coli*; the isolate of this cat was annotated in GenBank as *E. coli* 2008 and groups with a known human AIEC strain. GenBank numbers for sequences: strain 042 (FN554766), K12 (RefSeq: GCF_000005845), IAI39 (NC_011750), Sakai (NC_002695), 576-1 (AJWS00000000), CFT073 (CP051263), UTI89 (CP000243), E22 (NZ_AAJV00000000.2), E24377A (CP000800), UMN026 (NC_011751), KD2 (NZ_AJWP00000000).

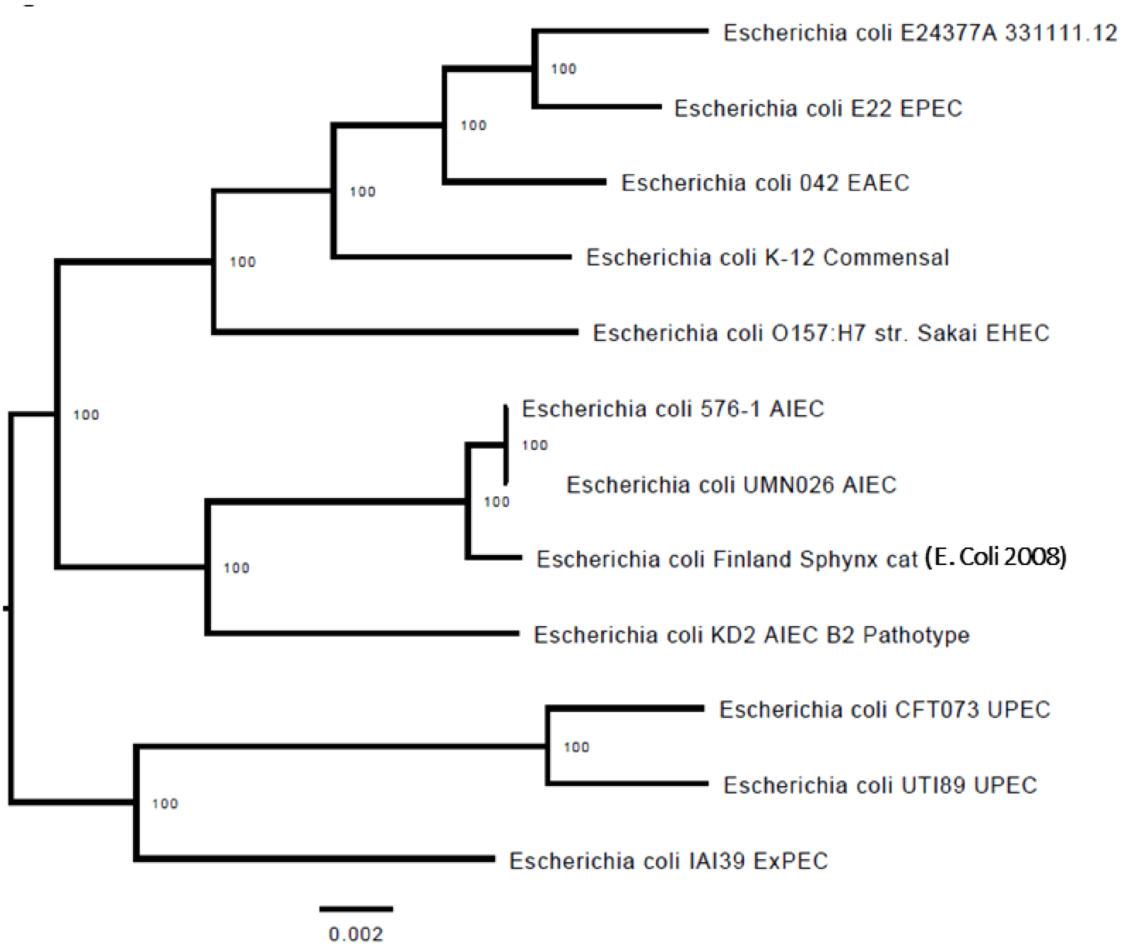

Supplemental Materials 1

a. Immunohistochemistry (IHC) methods

For IHC, primary antibodies included anti-Iba-1 (rabbit polyclonal, 1:500, O19-19741, FUJIFILM Wako Pure Chem. Corp) for macrophages, anti-CD45R (rat monoclonal, 1:600, MCA1258G, BIO-RAD) for B-lymphocytes, CD3 (rabbit polyclonal, 1:200, A0452, Dako) for T-lymphocytes, and feline coronavirus (FCoV) (mouse monoclonal, 1:600 dilution, MCA2194, BIO-RAD) for feline coronavirus. All primary antibodies were diluted in Normal Antibody Diluent (BD09, ImmunoLogic).

IHC for Iba-1 was performed using VectaStain ABC Peroxidase-kit (PK-4000, VectorLabs) and a secondary biotinylated goat anti-rabbit antibody (BA-1000, VectorLabs). For CD3, CD45, and FCoV, the BrightVision 2 step polymer detection system (anti-mouse/rabbit HRP, DPVBHRP, ImmunoLogic) was used. Briefly, sections were deparaffinized and rehydrated by routine methods. Tissue sections were incubated in 3% hydrogen peroxide for 10 minutes (CD3, CD45R, and FCoV) or 30 minutes (Iba-1) at room temperature to block endogenous peroxidase activity. Antigen retrieval was performed with citric acid (0.01 M, pH 6) at 99 °C for 20 minutes. Primary antibodies were incubated at room temperature for 60 minutes. Immunoreactions were visualized with 3, 3'-diaminobenzidine (DAB; BrightDAB, BS04, ImmunoLogic) and counterstained with Harris hematoxylin (1.09253, Merck KGaA). Positive controls included canine brain for Iba-1, canine tonsil and feline spleen for CD3 and CD45R, respectively, and feline lymph node from a diagnosed FIP case for FCoV. Negative control slides were incubated with diluent with omission of the primary antibody.

b. FCoV RT-PCR

Nucleic acids were extracted from the paraffin embedded scrolls of brain and spleen using xylene and the MagMax CORE Nucleic Acid Purification Kit, complex workflow (ThermoFisher Scientific, Waltham, MA, USA) on a KingFisher Flex Purification System (ThermoFisher Scientific). VetMAX Xeno Internal Positive Control RNA (ThermoFisher Scientific) was added to the sample prior to extraction to confirm no inhibition in the PCR assay. Primers and probe for the real-time RT-PCR assay were previously published by Gut et al.,1999 (see full reference below). The assay was performed in a 7500 Fast System (ThermoFisher Scientific) using TaqMan Fast Virus 1-Step Master Mix (ThermoFisher Scientific) and 0.4 µM of each primer, 0.2 µM vM of probe, and 1 µL of VetMAX Xeno Liz Assay (TheroFisher Scientific) in a 25 µL total volume reaction containing 7 µL of extracted nucleic acids. Cycling conditions were 50ºC for 5 minutes, 95ºC for 20 seconds, followed by 40 cycles of 95ºC for 3 seconds and 60ºC for 30 seconds.

Reference: Gut M, Leutenegger CM, Huder JB, Pedersen NC, Lutz H. One-tube fluorogenic reverse transcription-polymerase chain reaction for the quantitation of feline coronaviruses. J Virol Methods 1999;77:37-46.

c. *In situ* hybridization (ISH)

In situ hybridization (ISH) was performed using RNAscope® technology (Advanced Cell Diagnostics, Newark, CA) with a probe targeting *E. coli 16S* rRNA (B-E.Coli-*16S*rRNA,

cat. 433291) and RNAscope 2.5 HD Reagent Kit-Red (cat. 322350). The protocol used strictly followed the manufacturer's protocol without any modifications.

Internal ISH method positive control included formalin-fixed paraffin-embedded (FFPE)sample from mouse liver and the probe mouse Mm-Ppib (catalog number 313911, ACDbio). Positive control for *E. coli* included an FFPE sample of canine ileum from a case in which *E. coli* was cultured from the ileum and FISH was positive for *E. coli* (Langford Vets Diagnostic Laboratories, Bristol, UK) in the same FFPE block. Non-specific tissue hybridization was tested by using the probe mouse Mm-Ppib (catalog number 313911, ACDbio) on FFPE samples from the sphynx cat. Non-specific bacterial hybridization was tested by including an FFPE sample from the intestine of a dog with gram-positive rods and an FFPE sample from the nasal mucosa of a dog with gram-positive coccoid bacteria. In addition, the gram-positive rods described in the intestine of the sphynx cat were negative for ISH (internal tissue control).

d. Methods for virulence factors, *FimH* allele, and in silico group determination of the E. coli isolated from the sphynx cat

Virulence Finder 2.0 and FimTyper-1.0 (Center for Genomic Epidemiology) were used to detect virulence genes and to determine *FimH* allele, respectively. Clermon Typing, which also includes Mash group analysis, was used for *in silico* phylogroup determination. A phylogenetic tree was developed using known *E. coli* pathotypes (Phylogenetic Tree Building, Patric v. 3.6.12). Alignment was performed with MAFFT and the tree was built with branch support by RAxML Fast Bootstrapping.
